# SELECT: Glucagon-like peptide-1 receptor agonist in obese patients with cardiovascular disease in the absence of diabetes

**DOI:** 10.21542/gcsp.2024.26

**Published:** 2024-08-01

**Authors:** Susy Kotit, Marina Sous

**Affiliations:** 1 Aswan Heart Centre, Aswan, Egypt

## Abstract

Introduction: Obesity is a global epidemic affecting 2.5 billion people and is recognized as the fourth leading cause of global mortality. Obesity is characterized by excessive accumulation of body fat and is associated with a range of health consequences, including an elevated risk of cardiovascular disease (CVD). Glucagon-like peptide-1 (GLP-1) receptor agonists have been proven to reduce cardiovascular outcomes in patients with diabetes.

Study and results: The SELECT trial was a multicenter, double-blind, randomized, placebo-controlled, event-driven superiority trial, conducted at 804 clinical sites in 41 countries. Patients were randomly assigned in a 1:1 ratio, to receive once-weekly subcutaneous semaglutide at a dose of 2.4 mg or placebo. The primary cardiovascular efficacy endpoint was a composite of death from cardiovascular causes, nonfatal myocardial infarction, or nonfatal stroke, assessed in a time-to-first-event analysis. A total of 17,604 patients were recruited, with a mean age of 61.6 years and 72.3% males. The mean duration of exposure to semaglutide or placebo in the overall trial population was 34.2 ± 13.7 months. The primary CVD endpoint occurred in 6.5% (*n* = 569) of the semaglutide group and 8% (*n* = 701) in the placebo group (hazard ratio, 0.80; 95% CI, 0.72 to 0.90; *P* < 0.001). There was also a significant reduction of 9.39% in body weight among the patients taking semaglutide over 104 weeks after randomization versus 0.88% among the placebo group.

Conclusions: Semaglutide reduces the incidence of death from cardiovascular causes, nonfatal myocardial infarction, or nonfatal stroke in obese and overweight non-diabetic patients with preexisting cardiovascular disease.

## Introduction

Obesity has become a global epidemic affecting people across different age groups, posing a significant global health concern. In 2022, 2.5 billion adults were classified as overweight, with 35.6% falling under the obese category^1,2^. Alarmingly, there are more than 504 million women, 374 million men, and a staggering 159 million children and adolescents (aged 5–19 years) living with obesity^3–5^. Over the past few decades, adult obesity rates have more than doubled, and adolescent obesity has quadrupled since 1990^2^. Approximately, 1 in 8 people globally are now living with obesity^1,2^. The prevalence of obesity has significantly increased in nearly all countries globally, indicating a continuing and concerning trend^3,4^.

Obesity is recognized as the fourth leading cause of global mortality, resulting in 4.7 million deaths annually^6^. In 2019 an estimated 10% of global deaths were attributed to obesity-related consequences, representing a doubling of the proportion since 1990^7^. Consequently, obesity has emerged as one of the leading causes of death worldwide^7^.

Body mass index (BMI) is the current metric for assessing obesity. It is calculated as weight (in kilograms) divided by height in meters, squared^2^. According to the World Health Organization (WHO), overweight is defined as BMI ≥ 25 kg/m^2^ and obesity as BMI ≥ 30 kg/m^2^. Notably, each 5 kg/m^2^ increase in BMI above the range of 22.5 to 25 kg/m^2^ is associated with a 30% rise in overall mortality^2,8^.

**Figure 1.**
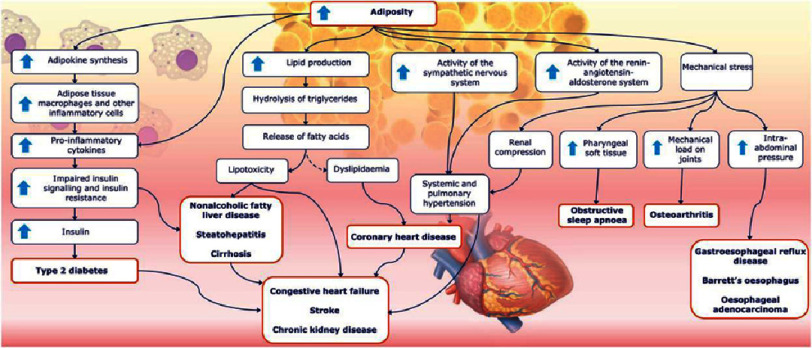
Risk factors and chronic conditions associated with adiposity. Common chronic diseases are shown in red boxes. The dashed arrow denotes an indirect association^14^.

Obesity is characterized by excessive accumulation of body fat. It is associated with a range of health consequences including an elevated risk of cardiovascular disease (CVD)^9–11^, alongside common cardiometabolic risk factors such as hypertension, type 2 diabetes, and dyslipidemia (Figure 1) serving as a major risk factor for CVD, which remains the leading cause of morbidity and mortality globally^9,12,13^.

Over the past two decades, deaths from obesity-related CVD have tripled, rising from 2.2 per 100,000 population to 6.6 per 100,000 population leading to 7.94 million deaths annually^11,15^. CVDs are the primary consequence of dietary risks including obesity and overweight. Weight loss and weight control are therefore essential interventions to effectively reduce overall CVD risk^16^. Modifying appetite using weight management medications represents a possible approach, potentially leading to significant (>10%) weight loss, which is clinically meaningful^6^.

**Figure 2.**
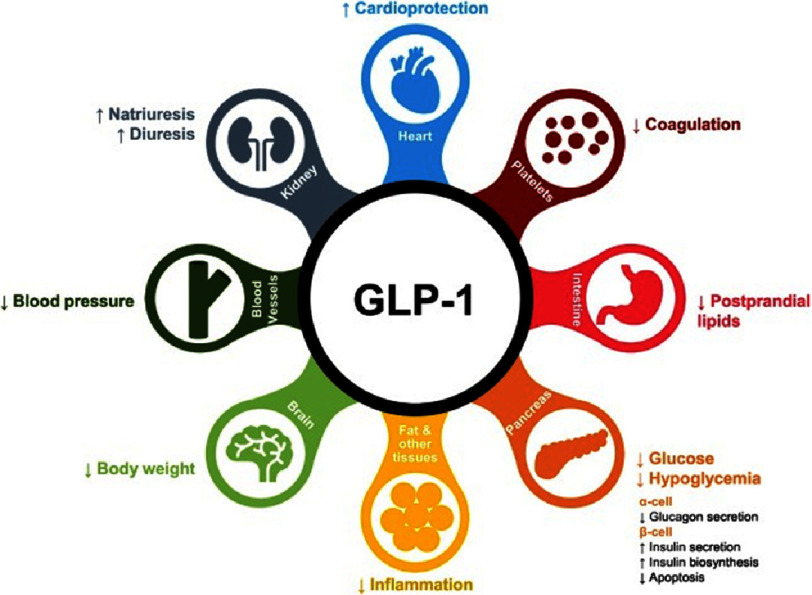
The effect of GLP-1 on different cell types/organs and its implications on cardiovascular disease^20,21^.

The glucagon-like peptide-1 (GLP-1) receptor agonist is among the six FDA-approved anti-obesity medications initially approved for type-2 diabetes management. GLP-1 is an incretin peptide hormone that works in a dose-dependent manner by stimulating insulin release and inhibiting glucagon secretion^17^. Additionally, It reduces appetite by slowing gastric emptying, leading to weight loss^18^. Furthermore, GLP-1 is associated with reduced inflammation, and improved energy homeostasis which is hypothesized to improve cardiovascular health. Importantly, GLP-1 was shown to reduce major adverse cardiovascular events (MACE) associated with type-2 diabetes in obese patients (Figure 2)^19^.

Semaglutide is a long-acting GLP-1 receptor agonist approved for the management of type-2 diabetes^22–24^ and overweight or obesity^25–27^. It has been shown to reduce body weight by a mean of 15.2% in overweight/obese patients without diabetes, but with the presence of at least one weight-related comorbidity (hypertension, dyslipidemia, CVD)^28^.

However, despite these promising findings, there remains a gap in comprehensive evidence regarding the impact of pharmacological interventions targeting obesity on improved cardiovascular outcomes, particularly in non-diabetic patients^6^.

The “Semaglutide Effects on Cardiovascular Outcomes in People with Overweight or Obesity” (SELECT) trial was conducted to investigate the impact of adding semaglutide to standard care on the risk of major adverse cardiac events (MACE) among overweight or obese non-diabetic patients with preexisting CVD.

### The SELECT trial

The SELECT trial was a multicenter, double-blind, randomized, placebo-controlled, event-driven superiority trial, conducted at 804 clinical sites in 41 countries^21,29^.

#### Trial population

Patients were eligible for enrollment if they were ≥ 45 years old, had BMI ≥ 27 mg/m^2^, and had established CVD, such as previous myocardial infarction, previous stroke, or symptomatic peripheral arterial disease.

Exclusion criteria included a previous diagnosis of diabetes, a glycated hemoglobin level of ≥ 6.5% (48 mmol per mole) measured at screening, treatment with any glucose-lowering medication or GLP-1 receptor agonist within the previous 90 days, New York Heart Association class IV heart failure, end-stage kidney disease or dialysis, cardiovascular or neurologic event within 60 days, or if they planned to undergo coronary, carotid, or peripheral revascularization.

#### Intervention and management

Patients were randomly assigned with a centralized system in a double-blind manner and in a 1:1 ratio without stratification, to receive once-weekly subcutaneous semaglutide at a dose of 2.4 mg or placebo.

The starting dose of semaglutide was 0.24 mg once weekly, increased every 4 weeks (to once-weekly doses of 0.5, 1.0, 1.7, and 2.4 mg) until the target dose of 2.4 mg was reached after 16 weeks. In case of unacceptable adverse effects, the dose-escalation intervals were extended, the treatment was paused, or maintenance doses below the 2.4 mg per week target dose were used.

In case of planned pregnancy, pancreatitis, or calcitonin levels equal to or greater than 100 ng per liter, semaglutide or placebo were discontinued.

Evidence-based recommendations were followed in the choice of medical management of underlying CVD. Development of diabetes developed during the trial resulted in the continuation of the assigned trial product with the use of glucose-lowering medication, even though the initiation of open-label treatment with a GLP-1 receptor agonist was prohibited.

#### Endpoints

The primary cardiovascular efficacy endpoint was a composite of death from cardiovascular causes, nonfatal myocardial infarction, or nonfatal stroke, assessed in a time-to-first-event analysis.

Secondary endpoints, assessed in time-to-first-event analyses and tested in hierarchical order, included death from cardiovascular causes, a composite heart failure endpoint (death from cardiovascular causes or hospitalization or an urgent medical visit for heart failure), and death from any cause. Efficacy analysis was based on the intention-to-treat method.

## Results

The SELECT study recruited 17,604 patients with a mean age of 61.6 years, of whom 72.3% were males. The mean BMI was 33.3 ±5.0 and 70% of the patients were obese (BMI >30). Additionally, 66.4% of the participants had pre-diabetes (HbA1c 5.7−6.4%). Notably, 75% of patients had previously experienced a myocardial infarction, 25% had a history of heart failure and most of the patients were taking anti-lipid (90.1%) and anti-platelet medications (86.2%).

Patients were randomly assigned in a 1:1 ratio to receive either semaglutide (*n* = 8803) or placebo (*n* = 8801). The mean duration of exposure to semaglutide or placebo in the overall trial population was 34.2 ±13.7 months (33.3 ±14.4 and 35.1 ±13.0 months, respectively).

**Figure 3.**
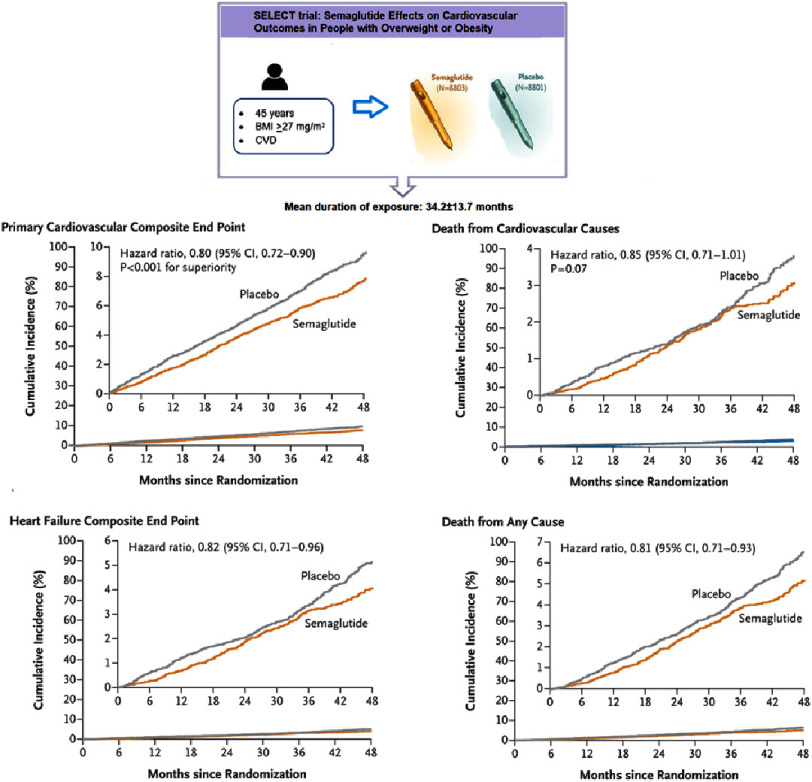
SELECT trial design and endpoints results.

The primary CVD endpoint occurred in 6.5% (*n* = 569) of the simaglutide group and 8% (*n* = 701) in the placebo group (hazard ratio, 0.80; 95% CI, 0.72 to 0.90; *P* < 0.001) (Figure 3).

The occurrence of the first confirmatory secondary endpoint, death from cardiovascular causes, was observed in 2.5% (*n* = 233) of the semaglutide group and 3.0% in the placebo group (hazard ratio, 0.85; 95% CI, 0.71 to 1.01; *P* = 0.07). Superiority testing was not conducted for the rest of the secondary endpoints because the difference in cardiovascular deaths between groups did not reach the required *p*-value for hierarchy testing.

Trial discontinuation occurred in 16.6% of the semagalutide group due to adverse events including gastrointestinal (10%), nervous system (1.4%), and metabolism and nutrition (1.2%) disorders compared to 8.2% in the placebo group.

Serious adverse events were reported in 33.4% of the semaglutide group compared to 36.4% in the placebo group.

Importantly, there was a significant reduction of 9.39% of body weight among the patients taking semaglutide over 104 weeks after randomization *versus* 0.88% among the placebo group (estimated treatment difference, −8.51 percentage points; 95% CI, −8.75 to −8.27).

## Discussion

The SELECT trial shows a clinically significant benefit associated with once weekly, subcutaneous 2.4 mg semaglutide treatment compared to placebo in overweight/obese patients with previous CVD and without diabetes. The trial resulted in a 20% reduction in the risk of cardiovascular outcomes and a 17% relative risk reduction for mortality, indicating the beneficial use of semaglutide for the secondary prevention of cardiovascular events. Furthermore, the differences in risk are evident early in the study, indicating a prompt risk reduction with semaglutide.

The mechanisms underlying cardiovascular risk reduction extend beyond weight loss and glycemic control. Emerging evidence points toward additional anti-inflammatory effects associated with semaglutide by suppressing IL-6 and TNF- *α* - pro-inflammatory cytokines^30^. These anti-inflammatory effects result in pro-thrombotic and anti-atherosclerotic benefits which play a crucial role in cardiovascular health, potentially impacting cardiac remodeling^31^.

In addition to the cardiovascular outcomes, the study assessed changes in body weight and waist circumference, in which semaglutide showed a 9.39% body weight reduction compared to 0.88% in the placebo arm at week 104. A more recent study that used the SELECT trial participants to examine the effect of semaglutide weight and anthropometric measures, showed that semgalutide demonstrated a significant mean weight reduction of 10.2% at week 208 compared to only 1.5% of the placebo group, sustained over 4 year^32^. In addition, it showed that semaglutide led to 7.7 cm in waist circumference reduction, compared to 1.3 cm in the placebo group ^32^.

However, the weight loss rate in the SELECT trial is significantly lower than the 15% weight loss reported in the previous “Semaglutide Treatment Effect in People with obesity” (STEP) trials in patients without diabetes^28,33^. This could be attributed to patient characteristics, age, and gender distribution. Another reason for this difference could be the objective of the studies, the SELECT trial focused on cardiovascular outcomes, and unlike the STEP trial, patients did not enroll for weight loss purposes^28^. Despite achieving only a 9% weight reduction, semaglutide is still more effective than older anti-obesity pharmacological treatments (such as orlistat, naltrexone-bupropion, and lorcaserin) ^34^.

The safety assessment demonstrated a lower occurrence of serious adverse events in the semaglutide group compared to placebo. However, a higher discontinuation rate during the early trial period (within 6 months) was observed, possibly due to the gastrointestinal symptoms during the first period of drug administration.

The SELECT was a randomized controlled trial, the gold standard for assessing causality, and included a large sample size, enhancing statistical power. However, it has limited generalizability, which arises from the predominantly Caucasian patient population that may not fully represent the global diversity of patients. Additionally, only 12.5% of the participants were African American, which raises concerns about applicability to this racial group.

Furthermore, the male predominance (with only 27.7% females) may not adequately address sex-specific effects. In addition, the study’s exclusive focus on preexisting cardiovascular disease limits its relevance to primary prevention or risk factors in otherwise healthy individuals.

In the Middle East and North Africa (MENA) region, the age-standardized rate of coronary heart disease (CHD) prevalence is the highest globally^35^. Notably, the region faces a high obesity rate (21.17%), and approximately 30.3% of CHD-related deaths in 2019 were attributed to a high BMI^35^. In this context, semaglutide, can play a crucial role in managing, promoting weight loss, and reducing cardiovascular risk in patients with existing heart disease. However, more evidence-based recommendations are needed in this specific population, which is significantly different from the study cohort.

In summary, the SELECT trial provides evidence suggesting that semaglutide, a long-acting GLP-1 receptor agonist, could have broad applicability in the secondary prevention of cardiovascular events, thereby holding significant potential for public health impact.

This trial highlights the groundbreaking role of GLP-1 receptor agonists, traditionally used for managing diabetes, in reducing cardiovascular risk among individuals with overweight and obesity, even in the absence of diabetes. Considering the escalating global prevalence of overweight and obesity, the observed decline in cardiovascular events–and concurrent substantial weight loss–position semaglutide as a promising therapeutic choice in addressing the intricate relationship between metabolic and cardiovascular well-being within this high-risk demographic. Nevertheless, there is a need to ascertain whether treatment with semaglutide provides cardioprotective and preventive benefits in patients without preexisting CVD. Further scientific investigation and real-world observations will be crucial in comprehending the long-term effects, safety profile, and broader applicability of semaglutide across diverse populations.

## Lessons learned

In obese and overweight patients with CVD but without diabetes, once-weekly semaglutide injections are superior to placebo in reducing the incidence of death from cardiovascular causes, nonfatal myocardial infarction, or nonfatal stroke (during a mean follow-up of 39.8 months).
